# Primary care health screening in patients with severe mental illness: What influence do financial incentives have?

**DOI:** 10.1371/journal.pmen.0000185

**Published:** 2025-05-30

**Authors:** Eugenia Romano, Ruimin Ma, Giovanni Sala, Mark Ashworth, Gayan Perera, Robert Stewart, Brendon Stubbs

**Affiliations:** 1 King’s College London, London, United Kingdom; 2 University of Liverpool, London, United Kingdom; 3 South London and Maudsley NHS Foundation Trust, London, United Kingdom; University of Liverpool, UNITED KINGDOM OF GREAT BRITAIN AND NORTHERN IRELAND

## Abstract

The Quality-of-Care Framework (QOF) aims to improve patient care for at risk groups through financial incentivisation. This study assesses the effect of changes in incentivisation of four health indicators for people with severe mental illness (SMI) on their recording versus controls. 9,250 patients with SMI aged >18 from South London and 12,729 controls were included using linked primary and mental healthcare records between 2006–2020. Mixed effect logistic regression controlling for age, gender, and neighbourhood deprivation estimated effects of incentivisation on health indicators in SMI and controls, and compared periods with/without incentivisation within the SMI sample and between SMI diagnostic groups. SMI patients overall were more likely than controls to be checked for all health indicators, and incentivisation was associated with increases in all screening measures in SMI compared to controls. In SMI patients, compared to pre-incentivisation, the likelihood of being checked increased overall (ranging from OR = 1.48 *p* < .001 for blood pressure to OR = 3.70 *p* < .001 for alcohol consumption); for alcohol consumption, however, this likelihood dropped significantly to lower odds when de-incentivised compared to the pre-incentivisation period (OR = 0.83 *p* < .001). Impacts were similar across SMI categories. While primary care financial incentivisation is associated with improved health screening in adults with SMI, its duration can impact quality of care, raising concerns over health inequalities in people with SMI and on the effectiveness of incentivised performance in healthcare.

## 1. Background

Severe mental illness (SMI) is an umbrella term encompassing a series of conditions including schizophrenia spectrum disorders, bipolar disorder and major depression [1]. People with SMI experience a higher premature mortality rate and poorer physical health compared to the general population [2,3]. Hypertension, cardiovascular disease, hepatitis, obesity, respiratory diseases and diabetes are among the most prevalent reported chronic illnesses in this population [4–6]. Further to impacting individuals’ quality of life and health, complex physical conditions are likely to be associated with a worsening course of SMI [2,7].

To compound matters, there are concerns that people with SMI receive inferior medical care and experience diagnostic shadowing, with clinicians attributing their symptoms to their mental health condition instead of running further health checks [2,8,9]. The reasons for this are complex but may include severity of SMI symptoms, the experience and perceived difficulties of clinicians, quality of treatment, as well as potential discriminatory attitudes from the healthcare personnel [10].

The majority of physical health care is provided in primary care. Therefore, any potential diagnostic shadowing in this location could have substantial negative effects on people with SMI [11]. In the UK, there is poor relational continuity in primary care for people with SMI, and informational and cross boundary continuity of care is also scarce [12,13]. A key role of primary care is to screen for physical illness and refer patients to proactive preventive health programs such as smoking cessation, physical activity classes and weight management services.

In the UK, the Quality and Outcomes Framework (QOF) is a programme first introduced in 2004 [14]. It aimed to reward general practitioners (GPs) in England for the quality of care provided by aligning financial incentives with the achievement of healthcare targets such as checking specific health checks (e.g., blood pressure) upon visiting the patient, with a specific range of health checks recommended according to the patient’s profile [14]. Previous research has investigated GP QOF achievements and found that they can help preventing cardiovascular risk factors [15]. However, certain targets have been de-incentivised over the years, either due to cost-effectiveness [16] with studies declaring that the QOF program costs too much for the yearly benefits it gives, more than what is usually considered good value by the NHS [17]. A systematic review on pay for performance healthcare schemes, in fact, found that the evidence on their effectiveness is mixed: the impact of these programmes is highly sensitive to features such as the size and type of financial incentives, the nature of performance measures, and the mechanisms for allocating rewards, and a poor design or a misalignment with clinical priorities may hence grant little improvement or even unintended negative consequences, especially considering the issue of long-term sustainability [18].

This intermittent removal and reintroduction of incentivised health indicators has caused serious concern over the impact on patients’ healthcare. For instance, studies like that of Minchin and colleagues [19] observed a decline in care quality following the withdrawal of financial rewards, highlighting the sensitivity of healthcare delivery to QOF incentives; it is however worth noting that the follow-up in the study was relatively short, and the initial drop in performance after de-incentivisation could reflect short-term adjustment, with a potential recovery over a longer period once clinicians adapt new care strategies. For people with SMI, the specific recommended health check indicators include smoking status (introduced for SMI population in 2008) [20], BMI, blood pressure, and alcohol consumption (all three first introduced in 2012). These health checks were introduced to close the physical health gap and incentivise healthcare attention on this population, but BMI incentivisation was discontinued between 2015 and 2019, and alcohol consumption incentivisation was discontinued from 2020. This has caused severe concerns over the healthcare quality for people with SMI, as this group is already often underserved by healthcare services [2,8,9]. An article by Matias and colleagues found a change in uptake for the physical health checks for BMI, cholesterol and alcohol consumption once they were removed from and re-introduced to the QOF list for people with SMI, suggesting that QOF incentives lead to better uptake of physical health checks [21]; however, no comparisons were made with pre-incentivisation periods nor with controls for the same kind of health checks. It thus remains unclear whether people with SMI receive more attention than the general population on the recommended health indicators, and how this has changed following de-incentivisation. Further light needs to be shed on the matter, as people with SMI must not risk a further loss of quality in the healthcare provided to them.

The aim of this study was to evaluate the impact of these financial incentive changes on the adherence to four health indicators (alcohol consumption, BMI, blood pressure, and smoking status) recommended for the SMI population, comparing their recording between SMI patients and controls in a period from 2006 to 2020. The outcomes of this study could inform ongoing debates and policy considerations regarding the reconfiguration of incentivized services within healthcare frameworks like QOF, aiming to optimize care for high-risk groups such as those with SMI.

## 2. Method

This is a longitudinal study relying on linked secondary administrative data to assess the effect of change of incentivisation in the recording of QOF indicators in SMI patients compared to controls. Below we have outlined details of the methodology for this study.

### 2.1 Data sources

Data were obtained from linked electronic health records (EHRs) from primary and secondary mental health care. Primary care data were obtained from Lambeth DataNet (LDN), a database containing data from general practices in the borough of Lambeth, south London, with more than 827,000 registered adult patients. LDN provides pseudonymised data such as socio-demographic information (e.g., age, ethnicity, gender and deprivation level), consultations, service referrals and medications [22]. Data from LDN have been linked to mental health care data from the South London and Maudsley NHS Foundation Trust (SLaM) Clinical Record Interactive Search (CRIS) [23]. SLaM is one of the largest mental health care providers in Europe, serving a wider geographic catchment of four boroughs in south London, but including the full borough of Lambeth covered by LDN. Data from structured fields in CRIS have been extensively supplemented by over 100 natural language process (NLP) applications detailed in an online catalogue [24]. CRIS has received full approval for secondary analysis (Oxford Research Ethnic Committee C, reference 23/SC/0257) and the SLaM Biomedical Research Centre (BRC) Case Register has been described in detail in previous publications [25,26]. The linkage between LDN and CRIS is regularly updated, and is curated by the SLaM Clinical Data Linkage Service (CDLS), within a local Trusted Research Environment [27]. Data were accessed in May 2022.

### 2.2 Study cohort

The SMI sample was drawn from patients with an active record in CRIS (active referral) or LDN (active registration), with a first-ever diagnosis in CRIS of psychotic, depressive, bipolar or other mental illness-related disorders diagnosed with a structured primary ICD-10 (International Classification of Disease, Tenth Revision) [28] diagnosis of F2x, F30x, F31x, F32.3 and F33.3 codes in CRIS, or a diagnosis in LDN of any disorders according to relevant Read and SNOMED Codes for SMI incentivisation according to the QOF [29,30]. Eligible patients were those aged 18 and over at the time of first SMI diagnosis who had a record in both CRIS and LDN between 1^st^ April 2006 and 1st March 2020. The sample of controls was drawn from patients registered in LDN with no SLaM contact and without any record of severe mental illness in LDN, registered within the same period as patients with SMI. Four controls were randomly selected and matched to each SMI patient based on gender, age (5-year age bands, from 18-22–88+), and the general practice where they had been registered for the longest time in the period covered by the study. Controls could be matched to more than one patient.

### 2.3 Primary outcomes

The primary outcome was a check for each recommended health indicator. The primary exposure was the effect of time (pre-incentivisation, incentivisation, and, for alcohol and BMI respectively, de-incentivisation and re-incentivisation) and the presence/absence of SMI. The recording of QOF-recommended health indicators was derived from LDN data, with one recording documented per year as either present or absent. Time was coded as dummy variables to obtain one for the pre-incentivisation period, one for the incentivisation period, and, for alcohol consumption and BMI, one for the de-incentivisation and re-incentivisation period respectively (2020 onwards as de-incentivisation for alcohol, 2015–19 for BMI with re-incentivisation after 2019).

### 2.4 Covariates

The model was adjusted for age, gender, and Index of Multiple Deprivation (IMD), which were extracted from CRIS. Age was calculated for each case based on year of birth and date of data extraction. Index of Multiple Deprivation (IMD), applied to Lower layer Super Output Area (LSOA), a standard administrative area with an average 1500 residents, was extracted from LDN and derived from source address fields. Patients’ diagnoses were divided in five groups: depressive disorders (for those cases involving depressive episodes as the primary feature), psychotic disorders (diagnosis involving primary psychotic symptoms), bipolar disorders, other non-specified affective disorders, and non-specified cases (see S1 File for details).

### 2.5 Statistical analysis

Analyses were performed with STATA 13 software [31]. Mixed effect logistic regression controlling for age, gender, and IMD were performed to measure overall odds of SMI patients of being checked on the health indicators, odds of the whole sample of being checked after the incentivisation, and interactions between SMI presence and different time periods (pre-incentivisation, incentivisation, and, for alcohol and BMI, de-incentivisation and re-incentivisation). Sensitivity analyses were also performed to see how odds of being checked on the health indicators varied across time periods in the SMI patient group, as well as to see how odds differed according to SMI sub-categories.

## 3. Results

**Fig 1:**
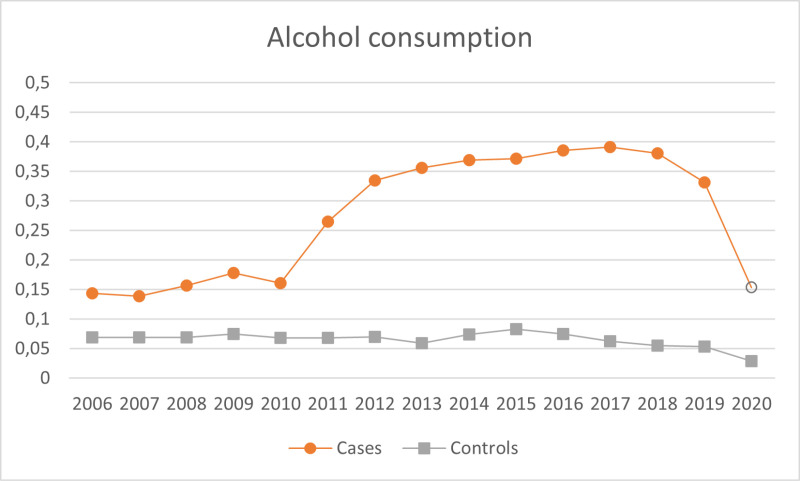
Proportion of participants checked on the alcohol consumption indicator across the years. Empty dots on the cases line indicate the years with de-incentivisation (2020).

**Fig 2:**
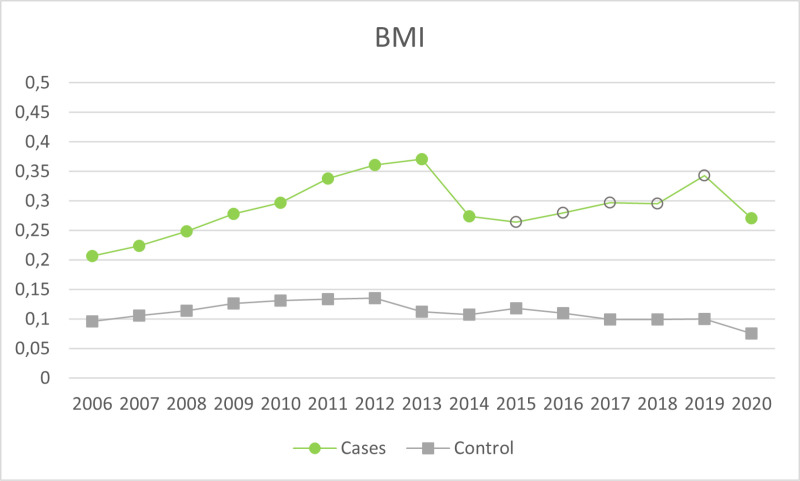
Proportion of participants checked on the BMI indicator across the years. Empty dots on the cases line indicate the years with de-incentivisation (2015-2019).

**Fig 3:**
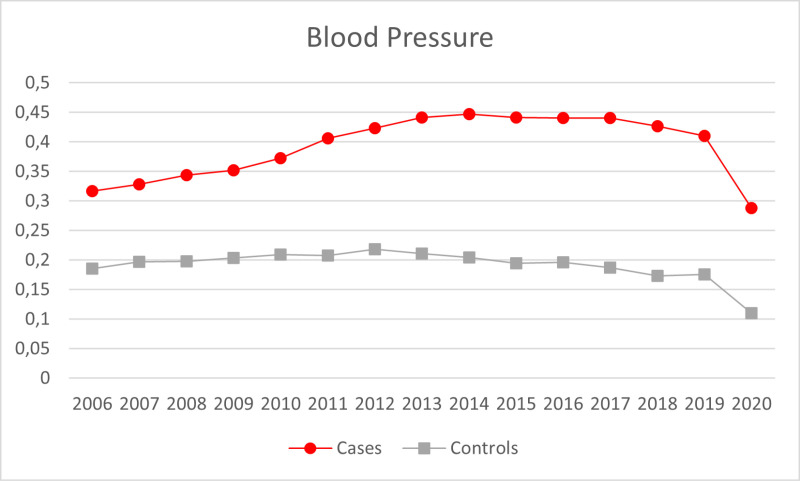
Proportion of participants checked on the blood pressure indicator across the years.

**Fig 4:**
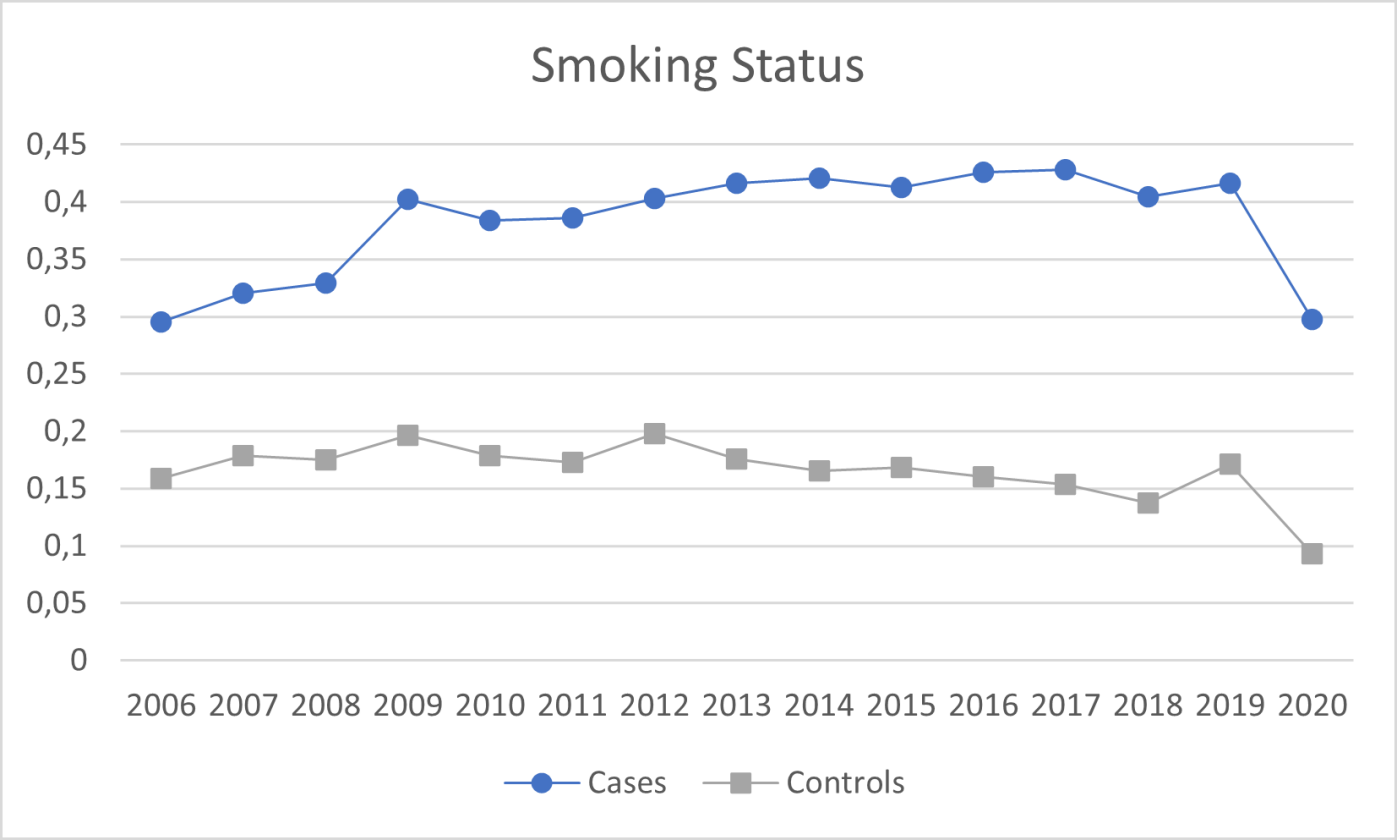
Proportion of participants checked on the smoking status pressure indicator across the years.

A total of 9,250 SMI adult patients were included, with a mean age of 54.2 (SD 17.6), 44.8% females, and a mean IMD score of 28.86 (SD 11.7). Of these, 6,361 (68.8%) had a psychotic disorder, 661 (7.2%) were diagnosed with a depressive disorder, 1,516 (16.4%) with bipolar disorder, 434 (4.7%) reported a diagnosis of other affective disorders, and 278 (3.01%) were non-specified. There were 12,729 controls, with a mean age of 56.93 (SD 17.7), 47.16% females, and a mean index of multiple deprivation of 28.96 (SD 11.7). The proportion of participants checked for each indicator across the years are further illustrated in Figs 1–4.

The results from the regression model are reported in Table 1. Overall, patients with SMI had higher odds of being checked for the recommended health indicators compared to controls (alcohol consumption OR = 3.44, SE = .12 *p* < .001, BMI OR = 3.79, SE = .12, *p* < .001, blood pressure OR = 3.80, SE = .13, *p* < .001, smoking status OR = 2.80, SE = .10, *p* < .001). This was confirmed by the time term and interactions, which revealed positive changes in all periods for SMI patients with negligible change in controls.

**Table 1:** Mixed effects logistic regression (whole sample).

	Patient Groupa	Incentivisation Periodb	SMI presence X Incentivisation periodb	SMI presence X De-incentivisation period b,c,d	SMI presence X Re-incentivisation periodb,d
Alcohol	3.44 (.12) ***	.92 (.02) ***	3.73 (.13)***	2.23 (.15) ***	
BMI	3.79 (.12) ***	1.05 (.03) *	1.46 (.05)***	1.30 (.05) ***	1.64 (.09)***
Blood Pressure	3.80 (.13) ***	.94 (.02) *	1.54 (.05) ***		
Smoking status	2.80 (.10)***	.99 (.02)	1.63 (.05) ***		

Values expressed as Odds Ratio (SE). Analysis controlled for age, gender, and index of multiple deprivation.

a = (controls as reference), b = (pre-incentivisation as reference), c = Alcohol consumption only, d = BMI only * = p =<.05, ** = p <=.01, *** = p =<.001

Table 2 reports analyses within the SMI sample investigating checks for health indicators at each time point, using pre-incentivisation as a reference. During incentivisation, the likelihood of being checked for the health indicator increased overall. In the case of BMI, it remained higher during the de-incentivisation period compared to pre-incentivisation (OR = 1.22 SE.02, *p* < .001) but had fallen to baseline levels during the re-incentivisation periods (OR = 1.03, SE.03). For alcohol consumption, the likelihood of being checked on the indicator dropped significantly to lower pre-incentivisation odds following de-incentivisation (OR =.83, SE.03, *p* < .001).

**Table 2:** Mixed effects logistic regression on the likelihood of being checked for each health indicator at different periods (SMI patients only).

	Incentivisation Period ^a^	De-incentivisation Period ^a^^,^^b^	Re-incentivisation Period ^a^^,^^b^^,^^c^
Alcohol	3.70 (.06) ^***^	.83 (.03) ^***^	
BMI	1.55 (.03) ^***^	1.22 (.02) ^***^	1.03 (.03)
Blood Pressure	1.48 (.02) ^***^		
Smoking status	1.65 (.03) ^***^		

Values expressed as Odds Ratio (SE). Analysis controlled for age, gender, and index of multiple deprivation.

^a^= (pre-incentivisation as reference),

^b^ = Alcohol consumption only,

^c^ = BMI only

* = p =<.05,

** = p <=.01,

*** = p =<.001

Table 3 reports sensitivity analysis for the likelihood of each SMI diagnosis of being checked for health indicators after incentivisation. Overall, all diagnostic groups reported greater likelihood of being checked for health indicators after the incentivisation, especially in case of alcohol, and odds ratios were generally similar in strength with the exception of those for the Other/ Not specified group, which only reported an increased likelihood for alcohol consumption and a lower likelihood of being checked in case of blood pressure and smoking status.

**Table 3:** Single mixed effects logistic regression on the likelihood of being checked for each SMI group after the incentivisation (SMI patients only).

	Psychotic Disorder	Depressive Disorder	Bipolar Disorder	Other Affective Disorder	Other/ Not Specified
Alcohol	3.87 (.07) ^***^	3.13 (.19) ^***^	3.91 (.16) ^***^	3.62 (.27) ^***^	1.56 (.13) ^***^
BMI	1.56 (.03) ^***^	1.49 (.10) ^***^	1.80 (.08) ^***^	1.38 (.12) ^***^	.84 (.09)
Blood Pressure	1.53 (.03) ^***^	1.39 (.07) ^***^	1.66 (.06) ^***^	1.18 (.07) ^**^	.60 (.05) ^***^
Smoking status	1.67 (.04) ^***^	1.64 (.12) ^***^	1.97 (.10) ^***^	1.31 (.11) ^**^	.71 (.08) ^**^

Values expressed as Odds Ratio (SE). Analysis controlled for age, gender, index of multiple deprivation, and time period (pre-incentivisation as reference).

* = p =<.05,

** = p <=.01,

*** = p =<.001

## 4. Discussion

To the best of our knowledge, this study is among the first to assess changes in primary care health screening measures in people with SMI in relation to incentivisation periods. While we found clear effects of incentivisation periods to increase receipt of screening, no screening measure exceeded 50% receipt in any time period. For the two screening measures where we could estimate the impact of de-incentivisation, the likelihood of being checked on alcohol consumption dropped rapidly to below pre-incentivisation levels, while BMI screening dropped by around 50%. BMI recording frequencies were falling at the time of re-incentivisation, but this evaluation period was limited in duration.

Previous research has reported how, in people with SMI compared to controls, the incentivisation of the QOF scheme for general physical health review for SMI patients led to better identification of cardiovascular risk factors [32]. This is a positive sign, considering the sizeable general health needs of this population [2,3], and it underlines the positive, intended effect of incentivisation in promoting much needed health screening. However, when looking at the likelihood of receiving a health check among SMI patients only, results for health indicators following de-incentivisation (i.e., removal of the incentive; BMI and alcohol consumption) suggest that incentivisation does not have substantial lasting impacts on practice. This is concerning, as research has found that attending the health checks is associated with reductions in disease risk in the general population [15]; at the same time, de-incentivisation of health checks can lead to an immediate decline in performance on quality measures [19]. This decline in screening provision may further reduce SMI patients’ access to adequate physical health checks, therefore delaying appropriate treatments and subsequently worsening physical health.

Our results also suggest that differences in incentivisation do not impact quality of care based on diagnosis groups. This is encouraging, as previous literature has reported how people with psychosis report quality of care can be inconsistent for them [33,34]. On the other hand, research has shown that socioeconomic deprivation is an important factor in explaining inequalities in quality of care for patients with psychosis [35], and since the catchment area for this study is high in levels of deprivation, this could have explained the lack of different incentivisation impacts across diagnoses groups.

Of course, when considering the de-incentivisation of health checks on alcohol consumption, it is important to take into account the effect of COVID-19. Alcohol consumption screening was de-incentivised for 2020, and the focus of healthcare during the pandemic crisis shifted heavily, so many of the routine checks could not be carried on anymore [36]. However, Fig 1 shows a diminishing likelihood of receiving this health check in the year immediately before the de-incentivisation and a similar decline is seen for BMI in Fig 2 from 2013 to 2014 prior to the discontinuation of the incentive in 2015. This could reflect anticipation: updated guidance for the QOF scheme is usually published in late March for the following next year (e.g., on 31^st^ of March for the 2023/24 period, [37]); this is to ensure that the updated guidance is available before the new financial year starts in April, and to give general practices enough time to review and implement the changes accordingly. Hence, it may be that if clinicians anticipate a de-incentivisation in one of the health indicators, their effort in recording them could drop already in the year before it is officially implemented.

This phenomenon could align with our earlier conclusion, that incentivisation only has an effect when it is present, and it may pose a serious issue in terms of quality of care for patients at high risk of health inequalities. This could also address the question of whether purely economic incentives are actually the best way to optimise healthcare practice, as while physical health screening initiatives might improve detection of risk factors, this does not necessarily translate into management of these risks [32]. There is a debate over whether economic incentives are actually beneficial for quality of care in healthcare settings, with scarce insights in patient experience and patient satisfaction, and frequent complaints over the effectiveness of this approach from patients themselves [38]. Future research should therefore investigate whether the likelihood of receiving a good quality of care according to the QOF scheme also translates into the probability of being referred to health prevention programmes. Integrating mental and physical healthcare through prevention would be the best avenue to tackle health inequalities afflicting people with SMI [39].

This study has several strengths, including a large, up-to-date sample, representative of its source population, characterised through a comprehensive list of recorded diagnoses thanks to data linkage between primary and specialist care databases. There are still important limitations to consider, such as the sample being restricted to a specific geographical area (the borough of Lambeth in South East London) which, while being a multi-ethnic urban population, it is also one with severe levels of deprivation and a relatively high prevalence of SMI, so our results may not necessarily generalise to other areas of London or the UK. Second, many covariates that could explain the relationship explored, such as social isolation, engagement, help-seeking behaviour, and general health, could not be taken in consideration in our model, and should hence be investigated in future studies.

## 5. Conclusions

In a large evaluation of the impact of incentivisation on primary care health screening in SMI, financial incentivisation for practices appears to improve the recommended health indicators but does not apparently have any longer-lasting improvement when these incentives are removed. Given the long-recognised substantial risks of premature mortality and multimorbidity in people with SMI, it is essential to investigate how quality of care might be improved for this patient group, particularly during periods when financial incentives are removed and when health screening might decline as a result.

## Supporting information

S1 File Division of ICD-10 and read/SNOMED coded diagnoses into categories used for the analysis (values from extraction). (DOCX)
