# Supplementary material for: Primary care health screening in patients with severe mental illness: What influence do financial incentives have?
Source: PLOS Ment Health. 2025 May 30;2(5):e0000185. doi: 10.1371/journal.pmen.0000185 (PMC12798617; doi:10.1371/journal.pmen.0000185)
Supplement: S1 File — (DOCX) [file pmen.0000185.s001.docx]

**Supplementary Material**

Division of ICD-10 and read/SNOMED coded diagnoses into categories used for the analysis (values from extraction)

| **Depressive** | |
| --- | --- |
| **ICD-10 diagnosis** | **Snomed/Read codes** |
| [X]Recurr severe episodes/major depression+psychotic symptom | Eu333 |
| [X]Endogenous depression with psychotic symptoms | Eu333 |
| [X]Severe depressive episode with psychotic symptoms | Eu323 |
| [X]Major depression, recurrent without psychotic symptoms | Eu332 |
| Recurrent major depressive episodes, severe, with psychosis | E1134 |
| [X]Post-schizophrenic depression | Eu204 |
| [X]Recurr depress disorder cur epi severe without psyc sympt | Eu332 |
| [X]Recurrent severe episodes of psychotic depression | Eu333 |
| [X]Single episode of major depression and psychotic symptoms | Eu323 |
| [X]Recurrent depress disorder cur epi severe with psyc symp | Eu333 |
| [X]Single episode of psychotic depression | Eu323 |
| Single major depressive episode, severe, with psychosis | E1124 |
| F20.4 - Post-schizophrenic depression | . |
| [X]Single major depressive episode, severe, with psychosis, psychosis in remission | Eu329 |
| F32.3 - Severe depressive episode with psychotic symptoms | . |
| F32.3 - Severe depressive episode with psychotic symptoms | . |
| F33.3 - Recurrent depress disorder cur epi severe with psyc symp | . |
| F33.3 - Recurrent depress disorder cur epi severe with psyc symp | . |
| Manic-depressive - now depressed | E115. |
| Psychotic reactive depression | E130. |
| Recurrent reactive depressive episodes, severe, with psychosis | Eu333 |
| Severe major depression with psychotic features | Eu328 |
| **Bipolar** | |
| **ICD-10 diagnosis** | **Snomed/Read codes** |
| Bipolar affective disorder | Eu31. |
| [X]Bipolar affect disorder cur epi mild or moderate depressn | Eu313 |
| [X]Bipolar affective disorder, current episode mixed | Eu316 |
| [X]Bipolar disorder, single manic episode | Eu30. |
| [X]Bipolar affective disorder, unspecified | Eu31z |
| Mixed bipolar affective disorder | E116. |
| Unspecified bipolar affective disorder, NOS | E117z |
| Unspecified bipolar affective disorder | E117. |
| H/O: manic depressive disorder | 146D. |
| Bipolar affect disord, currently manic,severe with psychosis | E1144 |
| Bipolar affect disord, now depressed, severe, no psychosis | E1153 |
| Bipolar affective disorder, current episode depression | E115. |
| Bipolar affective disorder, current episode hypomanic | Eu310 |
| Bipolar affective disorder, current episode manic | E114. |
| Bipolar affective disorder, currently depressed, in full remission | E1156 |
| Bipolar affective disorder, currently depressed, mild | E1151 |
| Bipolar affective disorder, currently depressed, moderate | E1152 |
| Bipolar affective disorder, currently depressed, NOS | E115z |
| Bipolar affective disorder, currently depressed, unspecified | E1150 |
| Bipolar affective disorder, currently manic, in full remission | E1146 |
| Bipolar affective disorder, currently manic, mild | E1141 |
| Bipolar affective disorder, currently manic, moderate | E1142 |
| Bipolar affective disorder, currently manic, NOS | E114z |
| Bipolar affective disorder, currently manic, severe, with psychosis | Eu312 |
| Bipolar affective disorder, currently manic, unspecified | E1140 |
| Bipolar disorder | E11.. |
| Bipolar disorder in full remission | E1176 |
| Bipolar disorder in remission | Eu317 |
| Bipolar I disorder | Eu318 |
| Bipolar II disorder | Eu319 |
| FH: Manic-depressive state | 1287 |
| Mixed bipolar affective disorder, moderate | E1162 |
| Mixed bipolar affective disorder, NOS | E116z |
| [X]Bipolar II disorder | Eu31y |
| [X]Other bipolar affective disorders | Eu31y |
| Family history of bipolar disorder | 1287 |
| Mixed bipolar affective disorder, mild | E1161 |
| Mixed bipolar affective disorder, severe | E1163 |
| Mixed bipolar affective disorder, NOS | E1161 |
| F31 - Bipolar affective disorder | . |
| F31.0 - Bipolar affective disorder, current episode hypomanic | . |
| F31.0 - Bipolar affective disorder, current episode hypomanic | . |
| F31.1 - Bipolar affect disord cur epi manic without psychotic symp | . |
| F31.1 - Bipolar affect disord cur epi manic without psychotic symp | . |
| F31.2 - Bipolar affect disorder cur epi manic with psychotic symp | . |
| F31.2 - Bipolar affect disorder cur epi manic with psychotic symp | . |
| F31.3 - Bipolar affect disorder cur epi mild or moderate depression | . |
| F31.3 - Bipolar affect disorder cur epi mild or moderate depression | . |
| F31.4 - Bipolar affect disord cur epi sev depres without psyc symp | . |
| F31.4 - Bipolar affect disord cur epi sev depres without psyc symp | . |
| F31.5 - Bipolar affect disord cur epi severe depres with psyc symp | . |
| F31.5 - Bipolar affect disord cur epi severe depres with psyc symp | . |
| F31.6 - Bipolar affective disorder, current episode mixed | . |
| F31.6 - Bipolar affective disorder, current episode mixed | . |
| F31.7 - Bipolar affective disorder, currently in remission | . |
| F31.7 - Bipolar affective disorder, currently in remission | . |
| F31.8 - Other bipolar affective disorders | . |
| F31.8 - Other bipolar affective disorders | . |
| F31.9 - Bipolar affective disorder, unspecified | . |
| F31.9 - Bipolar affective disorder, unspecified | . |
| Mixed bipolar affective disorder, unspecified | E1160 |
| Mixed bipolar I disorder | E11y3 |
| Psychosis and severe depression co-occurrent and due to bipolar affective disorder | Eu315 |
| Severe manic bipolar I disorder without psychotic features | E1143 |
| **Psychotic** | |
| **ICD-10 diagnosis** | **Snomed/Read codes** |
| Non-organic psychosis | E1... |
| [V]Personal history of schizophrenia | ZV110 |
| Schizophrenic disorders | E10.. |
| [X]Other nonorganic psychotic disorders | Eu2y. |
| Chronic paranoid psychosis | E121. |
| Schizophrenia | Eu20. |
| [X]Paranoid schizophrenia | Eu200 |
| Non-organic psychoses | E1... |
| F20 - Schizophrenia | Eu20. |
| Chronic schizophrenic | E1002 |
| Paranoid schizophrenia | E103. |
| [X]Affective psychosis NOS | Eu3z. |
| F20.0 - Paranoid schizophrenia | E103. |
| [X]Schizophrenia | Eu20. |
| [X]Unspecified nonorganic psychosis | Eu2z. |
| [X]Schizoaffective disorder, unspecified | Eu25z |
| Depressive psychoses | E11.. |
| [X]Brief reactive psychosis NOS | Eu23z |
| H/O: schizophrenia | 1464 |
| [X]Catatonic schizophrenia | Eu202 |
| H/O: psychosis | 146H. |
| [X]Catatonic stupor | Eu202 |
| [X]Chronic hallucinatory psychosis | Eu2y. |
| Reactive depressive psychosis | E130. |
| [X]Chronic undifferentiated schizophrenia | Eu205 |
| [X]Cotard syndrome | Eu222 |
| [X]Paranoid psychosis | Eu220 |
| [X]Simple schizophrenia | Eu206 |
| [X]Manic-depress psychosis,depressed type+psychotic symptoms | Eu333 |
| [X]Schizophrenia, schizotypal and delusional disorders | Eu2.. |
| [X]Reactive psychosis | E13.. |
| [X]Paranoia | Eu220 |
| [X]Paranoid state | Eu220 |
| [X]Paraphrenic schizophrenia | Eu200 |
| [X]Schizoaffective psychosis NOS | Eu25z |
| [X]Schizoaffective psychosis, depressive type | Eu251 |
| [X]Schizophreniform disord NOS | Eu20y |
| Unspecified schizophrenia | E1000 |
| [X]Schizophreniform psychosis, depressive type | Eu251 |
| [X]Schizophrenifrm psychos NOS | Eu20y |
| [X]Single episode of psychogenic depressive psychosis | Eu323 |
| Acute exacerbation of chronic paranoid schizophrenia | E1034 |
| Acute paranoid reaction | E133. |
| Acute polymorphic psychotic disorder co-occurrent with symptoms of schizophrenia | Eu231 |
| Acute polymorphic psychotic disorder without symptoms of schizophrenia | Eu230 |
| Acute schizo affective psychosis | E107. |
| Acute schizophrenia-like psychotic disorder | Eu232 |
| Acute schizophrenic episode | E104. |
| [X]Puerperal psychosis NOS | Eu531 |
| Acute transient psychotic disorder | Eu23. |
| Affective psychosis | E11z. |
| Other affective psychosis NOS | E11zz |
| [X]Acute and transient psychotic disorder, unspecified | Eu23z |
| [X]Dissociative [conversion] disorders | Eu44. |
| Other and unspecified manic-depressive psychoses | E11y. |
| F20.0 - Paranoid schizophrenia | E103. |
| Brief reactive psychosis | E13y1 |
| Catatonic schizophrenia | E102. |
| Catatonic schizophrenia NOS | E102z |
| Chronic paranoid schizophrenia | E1032 |
| Delusional disorder | Eu220 |
| Non-organic psychosis NOS | E1z.. |
| F20.1 - Hebephrenic schizophrenia | . |
| F20.2 - Catatonic schizophrenia | . |
| F20.3 - Undifferentiated schizophrenia | . |
| F20.5 - Residual schizophrenia | . |
| F20.6 - Simple schizophrenia | . |
| F20.6 - Simple schizophrenia | . |
| F20.8 - Other schizophrenia | . |
| F20.8 - Other schizophrenia | . |
| F20.9 - Schizophrenia, unspecified | . |
| F20.9 - Schizophrenia, unspecified | . |
| Unspecified affective psychoses NOS | E11z0 |
| F21 - Schizotypal disorder | . |
| F21.X - Schizotypal disorder | . |
| F22 - Persistent delusional disorders | . |
| Hypomanic psychoses | E110. |
| F22.0 - Delusional disorder | . |
| Schizotypal personality | E2122 |
| F22.0 - Delusional disorder | . |
| F22.8 - Other persistent delusional disorders | . |
| F22.8 - Other persistent delusional disorders | . |
| F22.9 - Persistent delusional disorder, unspecified | . |
| F22.9 - Persistent delusional disorder, unspecified | . |
| F23 - Acute and transient psychotic disorders | . |
| Acute hysterical psychosis | E131. |
| Manic psychosis | E110. |
| Other paranoid states NOS | E12y |
| F23.0 - Acute polymorphic psychot disord without symp of schizopha | . |
| F23.0 - Acute polymorphic psychot disord without symp of schizoph'a | . |
| F23.0 - Acute polymorphic psychot disord without symp of schizoph'a | . |
| F23.1 - Acute polymorphic psychot disord with symp of schizophrenia | . |
| F23.1 - Acute polymorphic psychot disord with symp of schizophrenia | . |
| F23.2 - Acute schizophrenia-like psychotic disorder | . |
| [X]Conversion hysteria | Eu44. |
| Reactive confusion | E132. |
| F23.2 - Acute schizophrenia-like psychotic disorder | . |
| F23.3 - Other acute predominantly delusional psychotic disorders | . |
| F23.3 - Other acute predominantly delusional psychotic disorders | . |
| F23.8 - Other acute and transient psychotic disorders | . |
| F23.8 - Other acute and transient psychotic disorders | . |
| F23.9 - Acute and transient psychotic disorder, unspecified | . |
| F23.9 - Acute and transient psychotic disorder, unspecified | . |
| Reactive psychoses | E13.. |
| F24 - Induced delusional disorder | . |
| F25 - Schizoaffective disorders | . |
| F25.0 - Schizoaffective disorder, manic type | . |
| F25.0 - Schizoaffective disorder, manic type | . |
| F25.1 - Schizoaffective disorder, depressive type | . |
| F25.1 - Schizoaffective disorder, depressive type | . |
| F25.2 - Schizoaffective disorder, mixed type | . |
| F25.8 - Other schizoaffective disorders | . |
| F25.8 - Other schizoaffective disorders | . |
| F25.9 - Schizoaffective disorder, unspecified | . |
| [X]Other acute and transient psychotic disorders | Eu23y |
| F25.9 - Schizoaffective disorder, unspecified | . |
| F28 - Other nonorganic psychotic disorders | . |
| [X]Induced psychotic disorder | Eu24. |
| F28.X - Other nonorganic psychotic disorders | . |
| F29 - Unspecified nonorganic psychosis | . |
| [X]Schizophrenia, unspecified | Eu20z |
| [X]Persistent delusional disorder, unspecified | Eu22z |
| F29.X - Unspecified nonorganic psychosis | . |
| [X]Capgras syndrome | Eu221 |
| Other specified non-organic psychoses | E1y.. |
| [X]Schizotypal personality disorder | Eu21. |
| Folie a deux | E123. |
| Hebephrenic schizophrenia | E101. |
| Hebephrenic schizophrenia NOS | E101z |
| Induced delusional disorder | Eu24. |
| Late paraphrenia | Eu220 |
| [X]Conversion reaction | Eu44. |
| Manic-depressive psychoses | E11.. |
| Manic-depressive psychosis | Eu31. |
| Mixed schizophrenic and affective pschosis | Eu252 |
| Other nonorganic psychoses | E13.. |
| Other paranoid states | E12y. |
| Other reactive psychoses NOS | E13yz |
| Other schizophrenia | E10y. |
| Paranoid disorder | E12.. |
| Paranoid psychosis | E12z. |
| Paranoid schizophrenia NOS | E103z |
| Paraphrenia | E122. |
| Persistent delusional disorder | Eu22. |
| Prodromal schizophrenia | Eu21. |
| Psychogenic paranoid psychosis | E134. |
| Psychogenic stupor | E13y0 |
| Psychotic | Eu2z. |
| Psychotic disorder | E13z. |
| Residual schizophrenia | E106. |
| Sander's disease | E121. |
| Schizoaffective disorder | Eu25. |
| Schizoaffective disorder, depressive type | Eu251 |
| Schizoaffective disorder, manic type | Eu250 |
| Schizoaffective disorder, mixed type | Eu250 |
| Schizoaffective schizophrenia | E107. |
| Schizoaffective schizophrenia in remission | E1075 |
| Schizo-affective schizophrenia NOS | E107z |
| Schizophrenia simplex | E100. |
| Schizophrenic psychoses | E10.. |
| Schizotypal personality disorder | Eu21. |
| Simple paranoid state | E120. |
| Simple schizophrenia | E100. |
| Undifferentiated schizophrenia | Eu203 |
| Unspecified manic-depressive psychoses | E11y0 |
| Unspecified paranoid schizophrenia | E1030 |
| **Other affective** | |
| **ICD-10 diagnosis** | **Snomed/Read codes** |
| [X]Mania with psychotic symptoms | Eu302 |
| [X]Manic episode | Eu30. |
| Manic-depressive illness | Eu31. |
| [X]Mania with mood-congruent psychotic symptoms | Eu302 |
| [X]Mania with mood-incongruent psychotic symptoms | Eu302 |
| [X]Other manic episodes | Eu30y |
| [X]Mania NOS | Eu30z |
| Single manic episode, severe, with psychosis | E1104 |
| Single manic episode, mild | E1101 |
| [X]Unspecified mood affective disorder | Eu3z. |
| [X]Manic episode, unspecified | Eu30z |
| F30 - Manic episode | . |
| F30.0 - Hypomania | . |
| F30.0 - Hypomania | . |
| F30.1 - Mania without psychotic symptoms | . |
| F30.1 - Mania without psychotic symptoms | . |
| F30.2 - Mania with psychotic symptoms | . |
| F30.2 - Mania with psychotic symptoms | . |
| F30.8 - Other manic episodes | . |
| F30.9 - Manic episode, unspecified | . |
| F30.9 - Manic episode, unspecified | . |
| Hypomania | Eu300 |
| Mania | Eu301 |
| Manic disorder, single episode | E110. |
| Single manic episode, severe | E1103 |
| Manic disorder, single episode NOS | E110z |
| Manic-depressive - now manic | E114. |
| Mood disorder | E11.. |
| Recurrent manic episodes | E111. |
| Recurrent manic episodes, unspecified | E1110 |
| Single manic episode, unspecified | E1100 |
| **Other / Not specified** | |
| **ICD-10 diagnosis** | **Snomed/Read codes** |
| On severe mental illness register | 9H8.. |
| [X]Severe mental and behavioural disorders associated with the puerperium, not elsewhere classified | Eu531 |
